# Heparin-coated *vs*. Non-coated Cardiopulmonary Bypass Circuits: Comparing Immediate Results with Different Target Activated Clotting Time

**DOI:** 10.21470/1678-9741-2019-0387

**Published:** 2020

**Authors:** Muhammet Onur Hanedan, Mehmet Ali Yürük, Ali Kemal Arslan, Aşkın Kılıç, Ufuk Sayar, İlker Mataracı

**Affiliations:** 1 Department of Cardiovascular Surgery, University of Health Sciences, Ahi Evren Thoracic and Cardiovascular Surgery Training and Research Hospital, Trabzon, Turkey.

**Keywords:** Cardiopulmonary Bypass, Heparin, Hospital Mortality, Length of Stay, Erythrocyte Transfusion, Silver, Cardiac Surgical Procedures, Intensive Care Units, Postoperative Period

## Abstract

**Objective:**

To compare immediate postoperative results in patients receiving heparin-albumin-coated and non-coated circuits.

**Methods:**

A total of 241 patients undergoing on-pump cardiac surgery were divided into two groups: those receiving heparin-coated circuits (Bioline®, Maquet Cardiopulmonary AG., Hirrlingen, Germany) and those receiving non-coated circuits (Maquet Cardiopulmonary AG., Hirrlingen, Germany).

**Results:**

Activated clotting times (ACT) during cardiopulmonary bypass (CPB) were significantly shorter in the heparin-albumin-coated group than in the non-coated group (355.64±34.12 *vs.* 560.38±90.20, respectively, *P*=0.001). In-hospital mortality and postoperative stroke rates and lengths of intensive care unit stay were similar between the groups; in contrast, in the heparin-albumin-coated group, patients had significantly better outcomes for hospital stay, drainage, and need for erythrocyte transfusion.

**Conclusion:**

Heparin-coated circuits and reduced level of systemic heparinization with 300 seconds of target ACT level in cardiac surgery under CPB are safe and result in a very satisfactory clinical course.

**Table:** 

Abbreviations, acronyms & symbols	
**ACT**	**= Activated clotting time**
**AVR**	**= Aortic valve replacement**
**CABG**	**= Coronary artery bypass grafting**
**COPD**	**= Chronic obstructive pulmonary disease**
**CPB**	**= Cardiopulmonary bypass**
**EuroSCORE**	**= European System for Cardiac Operative Risk Evaluation**
**ICU**	**= Intensive care unit**
**LV**	**= Left ventricular**
**MVR**	**= Mitral valve replacement**

## INTRODUCTION

Activation of the complement cascade, oxidative stress, and coagulation pathways induced by cardiopulmonary bypass (CPB) resulting in systemic inflammatory response syndrome after open heart surgery may cause several complications, like bleeding or organ dysfunctions^[1]^. Several CPB circuits with heparin-coated or surface-modifying agents are available. These systems have been shown to reduce inflammatory response and to provide better hemocompatibility.

To avoid circuitry blood clotting and thromboembolic complications, systemic heparin is administered to both the patient and the circuits^[2]^. Heparin is the most known anticoagulant used in CPB because of its rapid onset, effectiveness, ease of reversal by protamine, and low cost. While heparin is most known for its impact on the coagulation pathway, there is some evidence that heparin also affects fibrinolysis and platelet function independent of CPB. The effect on fibrinolysis and platelets may result in postoperative bleeding or increasing the transfusions^[3]^.

Heparin dosage for anticoagulation during CPB is calculated with an empiric formula based on the patient’s body weight and preoperative activated clotting time (ACT). Commonly for initiating CPB, ACT length must be > 480 seconds with non-heparin-coated circuits. Some authors have reported that this full-dose anticoagulation approach unnecessarily exposes the patient to excessive blood loss^[4]^. Heparin coating may decrease the appropriate ACT levels, which are resulting in less transfusion and bleeding.

In this study, we aimed to compare immediate postoperative results (*e.g*., drainage, erythrocyte transfusion, postoperative stroke, and mortality) in patients receiving heparin-albumin-coated and non-coated circuits.

## METHODS

We performed a retrospective database review between January 1^st^, 2015 and December 31^st^, 2016 and identified a total of 241 adult patients who underwent on-pump cardiac surgery. Emergency surgeries, minimal invasive procedures, and non-sternotomy patients were not included in the study. Patients were divided into two groups according to circuit coating property.

In our clinic, we were using non-coated circuits (Maquet Cardiopulmonary AG., Hirrlingen, Germany) before 2016 routinely, and by 2016 we started to use heparin-albumin-coated circuits (Bioline®, Maquet Cardiopulmonary AG., Hirrlingen, Germany). All perfusion tubing systems were completely coated, except for the cannulas.

Hemochron® Jr. Signature plus Whole Blood Microcoagulation System (Accriva Diagnostics, San Diego, California, United States of America) was used to measure ACT. This system uses silica, kaolin, and phospholipid as activators and measures the elapsed time between the start of the test and clot formation, and the ACT is automatically converted to a reference celite-based ACT value.

Before CPB, 300 IU/kg of heparin (Vasparin® Tekirdag, Turkey) was administered intravenously to patients receiving non-coated circuits. Readministration of 5000 IU heparin boluses took place if the ACT was < 480 seconds. A reduced dose of heparin (150 IU/kg) was administered for systemic anticoagulation to patients receiving heparin-albumin-coated circuits. Readministration of 2500 IU heparin boluses took place if the ACT was < 300 seconds. ACT was repeatedly determined during CPB, after protamine administration, and two hours postoperatively. Myocardial protection consisted of intermittent antegrade administration of cold blood cardioplegic solution. After completion of CPB, heparin was antagonized with protamine in a ratio of 1:1.

The amount of postoperative bleeding from the time of sternal closure until the drains were removed was recorded. Postoperative 24-hour drainage was used for analysis. Normovolemic anemia was accepted to a hematocrit level of 0.25 postoperatively; a level less than this was considered an indication for allogeneic red blood cell transfusion.

CPB was performed with a Terumo® Advanced Perfusion System 1 (Terumo Cardiovascular Group, Ann Arbor MI) with a non-pulsatile flow control and at a flow rate of 2.4 L/m^2^/min. Systemic moderate hypothermia (30 ˚C) was used in coronary and valve surgery; in aortic procedures, deep hypothermia (24 ˚C) was used. Standard adult sizes of the circuits were used and primed with 1000 mL of lactated ringer.

Antiplatelet therapy was not stopped before surgery. Vitamin K antagonist was stopped and changed to low-molecular-weight heparin before surgery.

### Statistical Analysis

Data were expressed as mean ± standard deviation for quantitative variables and as number and percentage for categorical variables. The groups were compared by Student’s *t*-test for continuous variables and the c^2^ or Fisher’s exact test for categorical variables. A *P*-value < 0.05 was statistically significant.

## RESULTS

The patients’ baseline demographic characteristics are summarized in Table 1 and they were comparable, except for a higher incidence of extracardiac arteriopathy in the heparin-albumin-coated group. Performed operations are mentioned in Table 2. The chief procedure was coronary artery bypass grafting (CABG), for 60% of the patients in the heparin-albumin-coated group and 83% in the non-coated group. Table 3 shows the operative and postoperative variables. In heparin-albumin-coated group, we observed significantly longer cross-clamp times. ACT during CPB is significantly shorter in the heparin-albumin-coated group than in the non-coated group (355.64±34.12 *vs*. 560.38±90.20, respectively, *P*=0.001) In-hospital mortality rates, postoperative cerebrovascular event rates, and lengths of intensive care unit (ICU) stay were similar between the groups; in contrast, patients in the heparin-albumin-coated group had significantly better outcomes for hospital stay, drainage, and need for erythrocyte transfusion.

**Table 1:** Patients' preoperative demographic and clinical characteristics.

	Group 1	Group 2	P-value
	Heparin-albumin-coated circuits (n=135)	Non-coated circuits (n=106)	
Age (years)	63.87±10.16	62.17±10.61	0.196
Logistic EuroSCORE II	4.52±4.44	4.51±4.97	0.987
Male patients	100 (74.1%)	79 (74.5%)	0.996
Body surface area (m^2^)	1.84±0.18	1.86±0.18	0.476
LV ejection fraction (%)	53.83±9.94	54.27±10.07	0.732
Smoking history	60 (44.4%)	48 (45.3%)	0.897
Diabetes mellitus	73 (54.1%)	57 (53.8%)	0.963
Hypertension	94 (69.6%)	67 (63.2%)	0.293
Creatinine	1.04±0.31	1.16±0.74	0.086
COPD	37 (27.4%)	29 (27.4%)	0.993
Extracardiac arteriopathy	29 (21.5%)	12 (11.3%)	0.037
Cerebrovascular disease	2 (1.5%)	2 (1.9%)	10.999
Atrial fibrillation	23 (17%)	16(15.1%)	0.684
Hematocrit (%)	40.89±4.98	41.17±4.44	0.642

COPD=chronic obstructive pulmonary disease; EuroSCORE=European System for Cardiac Operative Risk Evaluation; LV=left ventricular Data are expressed as mean ± standard deviation or as number and percentage. *P*<0.05 was considered statistically significant.

**Table 2:** Type of operation in groups.

	Group 1	Group 2
	Heparin-albumin-coated circuits (n=135)	Non-coated circuits (n=106)
CABG	81	88
MVR/Mitral repair	11	4
MVR+CABG	10	3
AVR	14	4
AVR+CABG	8	1
AVR+MVR	3	1
Aortic procedures (±AVR)	8	5
Redo surgeries	5	3

AVR=aortic valve replacement; CABG=coronary artery bypass grafting; MVR=mitral valve replacement

**Table 3:** Comparison of operative and postoperative results.

	Group 1	Group 2	*P*-value
	Heparin-albumin-coated circuits (n=135)	Non-coated circuits (n=106)	
Operation time (min)	219.67±53.34	222.97±47.20	0.616
Cross-clamp time (min)	58.29±22.49	51.67±23.56	0.027
CPB time (min)	90.78±29.75	84.25±29.45	0.091
ACT (sec) (during CPB)	355.64±34.12	560.38±90.20	0.001
ACT (sec) (after 2 hours)	103.58±2.71	104.24±3.69	0.125
Duration of mechanical ventilation (hours)	13.9±45.2	11.65±19.22	0.633
Intensive care unit stay (hours)	66.70±65.01	75.07±56.38	0.294
Drainage (mL)	529.11±267.97	660.75±279.73	0.001
Re-exploration for bleeding	3 (2.2%)	7 (6.6%)	0.111
Erythrocyte transfusion (U)	1.27±1.32	2.08±2.28	0.001
Postoperative stroke	3 (2.2%)	2 (1.9%)	0.998
30-day hospital death	3 (2.2%)	2 (1.9%)	0.998
Hospital stay (days)	4.48±3.25	9.97±6.88	0.027

ACT=activated clotting time; CPB=cardiopulmonary bypass Data are expressed as mean ± standard deviation or as number and percentage. P<0.05 was considered statistically significant.

## DISCUSSION

This study revealed similar clinical outcomes of non-coated and albumin-heparin-coated circuits in terms of in-hospital mortality rates, postoperative cerebrovascular event rates, and lengths of ICU stay. Furthermore, better outcomes for hospital stay, amount of mediastinal drainage, and need for erythrocyte transfusion were achieved with coated circuits. In fact, it is obvious that the proposal of maintaining CPB using heparin in a lesser amount with a shorter ACT and, at the same time, using bioartificial surfaces will have positive results. In this study, the rational basis of this hypothesis was investigated with literature examples.

Conventionally, an empirical dose of heparin has been used to inhibit coagulation for initiating and maintaining CPB to achieve an ACT level > 480 seconds. Achieving this target ACT level by giving high doses of heparin is associated with significantly higher postoperative blood loss^[5]^. A heparin titration method used in a study to achieve conventional ACT level resulted in using low doses of heparin, which is associated with lower blood loss^[6]^. In this recent study, we used low-dose heparin with titration model with a significantly lower ACT level.

Heparin-coated surfaces do not only reduce the systemic heparinization, they also reduce the systemic inflammatory process and oxidative stress^[7]^. Although the endpoint of our study is not to assay inflammatory responses, using more biocompatible surfaces is related with better outcomes. Tayama E. et al.^[8]^ showed reduced inflammatory response with heparin-coated circuits, but no benefits in clinical outcomes. In that study, heparin administration and target ACT levels were the same in both heparin-coated and non-coated groups. Similarly, there were no significant clinical outcomes in the study by Reser D. et al.^[1]^, in which three different biocompatible surface circuits were compared and heparin administration and target ACT levels were conventionally set. These consequences support our hypothesis.

Another important debate is that if low target ACT levels are safe or not. Ovrum E. et al.^[4]^ showed that a median ACT level (almost 300 seconds during CPB) was safe and resulted in a very satisfactory clinical course in 5,954 patients undergoing on-pump CABG with heparin-coated circuits^[4]^. In this recent study, mean ACT level in heparin-coated circuits was 355 seconds during the CPB and it was not associated with adverse clinical outcome.

Heparin coating the circuits increases cost and this technology is not routinely used for short-time devices due to higher initial costs^[9]^. Therefore, the advantages of this system, like reduction in blood loss and reduction of ventilator dependence and length of hospital stay, make heparin-coated circuits more reasonable to use in terms of overall costs^[10]^.

Limited use of anticoagulation during CPB and risk for stroke is another concern which was investigated previously, and stroke and mortality rates were comparable in heparin-coated circuits vs. conventional ones^[11]^. In our assay, we found no significant difference between the groups for 30-day hospital mortality and postoperative stroke rates, similarly to the literature.

A meta-analysis demonstrates parallel results, that biocompatible circuits have a limited effect (lower transfusion needs and atrial fibrillation rate) on morbidity, leading to shorter ICU and hospital stays^[12]^. A review by Mahmood S. et al.^[10]^ about heparin-bonded CPB circuit showed that perfusion with heparin-coated and heparin-polymer-coated bypass does not increase the risk of adverse effects but reduces blood loss, re-operation rates, ventilation time, length of ICU and hospital stays, and is also associated with improved biocompatibility.

### Limitations

The limitations of this study are its single-center nature, small sample size, and nonrandomized design. This study focused on immediate outcomes, so long-term follow-up data from randomized clinical trials will be needed to evaluate clinical outcomes.

## CONCLUSION

Comparable postoperative stroke and mortality rates were found in contrast with less blood transfusion, lower drainage, short periods of postoperative ventilator support, and reduced hospital stay seen in the study group. In conclusion, heparin-coated circuits and reduced level of systemic heparinization with a 300 seconds target ACT level in cardiac surgery under CPB are safe and result in a very satisfactory clinical course.

**Table:** 

Authors' roles & responsibilities	
MOH	Substantial contributions to the conception or design of the work; or the acquisition, analysis, or interpretation of data for the work; drafting the work or revising it critically for important intellectual content; agreement to be accountable for all aspects of the work in ensuring that questions related to the accuracy or integrity of any part of the work are appropriately investigated and resolved; final approval of the version to be published
MAY	Substantial contributions to the conception or design of the work; or the acquisition, analysis, or interpretation of data for the work; drafting the work or revising it critically for important intellectual content; agreement to be accountable for all aspects of the work in ensuring that questions related to the accuracy or integrity of any part of the work are appropriately investigated and resolved; final approval of the version to be published
AKA	Agreement to be accountable for all aspects of the work in ensuring that questions related to the accuracy or integrity of any part of the work are appropriately investigated and resolved; final approval of the version to be published
AK	Substantial contributions to the conception or design of the work; or the acquisition, analysis, or interpretation of data for the work; agreement to be accountable for all aspects of the work in ensuring that questions related to the accuracy or integrity of any part of the work are appropriately investigated and resolved; final approval of the version to be published
US	Drafting the work or revising it critically for important intellectual content; final approval of the version to be published
IM	Drafting the work or revising it critically for important intellectual content; agreement to be accountable for all aspects of the work in ensuring that questions related to the accuracy or integrity of any part of the work are appropriately investigated and resolved; final approval of the version to be published

## References

[1] Reser D, Seifert B, Klein M, Dreizler T, Hasenclever P, Falk V (2012). Retrospective analysis of outcome data with regards to the use of Phisio®-, Bioline®- or Softline®-coated cardiopulmonary bypass circuits in cardiac surgery. Perfusion.

[2] Teligui L, Dalmayrac E, Mabilleau G, Macchi L, Godon A, Corbeau JJ (2014). An ex vivo evaluation of blood coagulation and thromboresistance of two extracorporeal circuit coatings with reduced and full heparin dose. Interact Cardiovasc Thorac Surg.

[3] Lander H, Zammert M, FitzGerald D (2016). Anticoagulation management during cross-clamping and bypass. Best Pract Res Clin Anaesthesiol.

[4] Øvrum E, Tangen G, Tølløfsrud S, Skeie B, Ringdal MA, Istad R (2011). Heparinized cardiopulmonary bypass circuits and low systemic anticoagulation: an analysis of nearly 6000 patients undergoing coronary artery bypass grafting. J Thorac Cardiovasc Surg.

[5] Despotis GJ, Joist JH, Hogue CW Jr, Alsoufiev A, Kater K, Goodnough LT (1995). The impact of heparin concentration and activated clotting time monitoring on blood conservation. A prospective, randomized evaluation in patients undergoing cardiac operation. J Thorac Cardiovasc Surg.

[6] Shuhaibar MN, Hargrove M, Millat MH, O’Donnell A, Aherne T (2004). How much heparin do we really need to go on pump? A rethink of current practices. Eur J Cardiothorac Surg.

[7] Sohn N, Marcoux J, Mycyk T, Krahn J, Meng Q (2009). The impact of different biocompatible coated cardiopulmonary bypass circuits on inflammatory response and oxidative stress. Perfusion.

[8] Tayama E, Hayashida N, Akasu K, Kosuga T, Fukunaga S, Akashi H (2000). Biocompatibility of heparin-coated extracorporeal bypass circuits: new heparin bonded bioline system. Artif Organs.

[9] Voegele-Kadletz M, Wolner E (2011). Bio artificial surfaces - blood surface interaction. Mater Sci Eng.

[10] Mahmood S, Bilal H, Zaman M, Tang A (2012). Is a fully heparin-bonded cardiopulmonary bypass circuit superior to a standard cardiopulmonary bypass circuit?. Interact Cardiovasc Thorac Surg.

[11] Mangoush O, Purkayastha S, Haj-Yahia S, Kinross J, Hayward M, Bartolozzi F (2007). Heparin-bonded circuits versus nonheparin-bonded circuits: an evaluation of their effect on clinical outcomes. Eur J Cardiothorac Surg.

[12] Ranucci M, Balduini A, Ditta A, Boncilli A, Brozzi S (2009). A systematic review of biocompatible cardiopulmonary bypass circuits and clinical outcome. Ann Thorac Surg.

